# Vitamin B12 and Autism Spectrum Disorder: A Review of Current Evidence

**DOI:** 10.3390/nu17071220

**Published:** 2025-03-31

**Authors:** Mateusz Zwierz, Maria Suprunowicz, Katarzyna Mrozek, Jacek Pietruszkiewicz, Aleksandra Julia Oracz, Beata Konarzewska, Napoleon Waszkiewicz

**Affiliations:** Department of Psychiatry, Medical University of Bialystok, pl. Wołodyjowskiego 2, 15-272 Białystok, Poland; maria.suprunowicz@sd.umb.edu.pl (M.S.); 39913@student.umb.edu.pl (K.M.); 39929@student.umb.edu.pl (J.P.); aleksandra.oracz@sd.umb.edu.pl (A.J.O.); beata.konarzewska@umb.edu.pl (B.K.); napoleon.waszkiewicz@umb.edu.pl (N.W.)

**Keywords:** autism spectrum disorder, ASD, cobalamin, vitamin B12, supplementation, microbiome, dysbiosis

## Abstract

Vitamin B12 (cobalamin) plays a crucial role in neurodevelopment, particularly during pregnancy and early childhood. It is essential for DNA synthesis, red blood cell formation, and nervous system function. Maternal B12 levels are particularly important, as they influence fetal brain development. Inadequate maternal intake during pregnancy may lead to altered neurodevelopmental trajectories and increase the risk of ASD. Postnatally, insufficient dietary cobalamin in infants and young children could further contribute to cognitive and behavioral impairments. One potential mechanism linking low B12 levels to ASD involves its role in the gut microbiota balance. Dysbiosis, commonly observed in individuals with ASD, is associated with increased gut permeability, low-grade inflammation, and disruptions in the gut–brain axis, all of which may contribute to ASD symptoms. Additionally, B12 is essential for neurotransmitter metabolism, particularly in the synthesis of serotonin and dopamine, which regulate mood, cognition, and behavior. Cobalamin also plays a key role in neuronal myelination, which ensures efficient signal transmission in the nervous system. Disruptions in these processes could underlie some of the cognitive and behavioral features associated with ASD. Despite growing evidence, the link between B12 and ASD remains inconclusive due to inconsistent findings across studies. Research suggests that B12 levels may serve as a potential biomarker for disease progression and treatment response. However, many studies rely on single-time-point measurements, failing to account for individual variability, genetic predispositions, dietary intake, and environmental factors, all of which can influence B12 levels and ASD risk. Further longitudinal studies are needed to clarify this relationship.

## 1. Introduction

Although vitamin B12 (cobalamin) is synthesized primarily by plant organisms, its natural sources in the human diet are predominantly meat, eggs, and dairy products [1]. A deficit in this vitamin during pregnancy has been linked to an elevated risk of metabolic disorders in the mother, including obesity and insulin resistance. These conditions have the potential to negatively impact fetal development [2,3]. This underscores the importance of supplementation of this nutrient during key periods of a woman’s life. Health Canada guidelines, for prenatal nutrition, recommend a vitamin B12 intake of 2.6 µg per day for pregnant women and 2.8 µg per day for breastfeeding women [4].

Longitudinal research employing Mendelian randomization examined the influence of transcobalamin 2 (TCN2), a major transporter of vitamin B12, on the neurodevelopment of kids. The research indicated that women with a homozygous TCN2 variation had enhanced efficacy in vitamin B12 transport, resulting in a dose-dependent relationship between maternal meat intake and a decreased risk of behavioral impairments in their offspring [5]. These findings indicate that adequate maternal vitamin B12 levels, affected by food and genetic factors, may significantly influence long-term neurological health.

From a clinical point of view, the symptoms of severe and long-term cobalamin deficits and autism spectrum disorder (ASD) are similar. ASD is a neurodevelopmental disorder marked by challenges in social communication and interaction, as well as repetitive behavioral patterns. In both long-term cobalamin deficits and autism spectrum disorder, neurological symptoms may occur, including motor, cognitive, and mood-related impairments [6,7]. An increasing number of studies are indicating the possibility of a correlation between lower levels of vitamin B12 and the emergence of ASD [8,9].

This narrative review analyzes the latest literature on the relationship between vitamin B12 deficits and excess and the risk and development of autism. Both the results and limitations of available studies were taken into account to indicate directions for future research that could provide more conclusive answers about the role of this vitamin in the etiology and course of ASD.

## 2. Materials and Methods

Studies included were published between 2000 and 2024. The literature search was conducted on PubMed, Web of Science, and Google Scholar in December 2024, covering studies from 2000 to 2024 using relevant search terms, including “vitamin B12, cobalamin, ASD, autism spectrum disorder, gut microbiota, neurodevelopment, neuro-transmitters, inflammation, biomarkers, supplementation”. Both original research and review articles were considered during the initial screening. Mendeley Reference Manager Version 2.131.0 was used to remove duplicates that appeared due to overlapping search terms.

Since this work is a narrative review, no strict inclusion criteria were established for the selection of articles. Based on titles and abstracts, conference abstracts were excluded. Studies not written in English were also omitted. Additionally, supporting literature on potential therapeutic approaches was included. It is essential to note that the review is not systematic, and despite attempts to cover all studies, one should keep in mind significant limitations.

## 3. The Role of Vitamin B12 in Nervous System Development and Metabolism

Low levels of vitamin B12 have been demonstrated to suppress critical processes involved in the normal development of the fetal nervous system. These processes include reduced activity of methylation DNA, RNA, histones, and important enzymes necessary for myelin synthesis. These findings suggest that lower cobalamin levels may have adverse effects on fetal development, including neural tube defects and impaired neurological development [10]. A prolonged deficit in vitamin B12 can result in demyelination or damage to the myelin sheaths that surround nerve fibers in the brain and spinal cord. This can lead to the development of motor disorders, motor coordination problems, and impaired sensory function [11].

**Figure 1:**
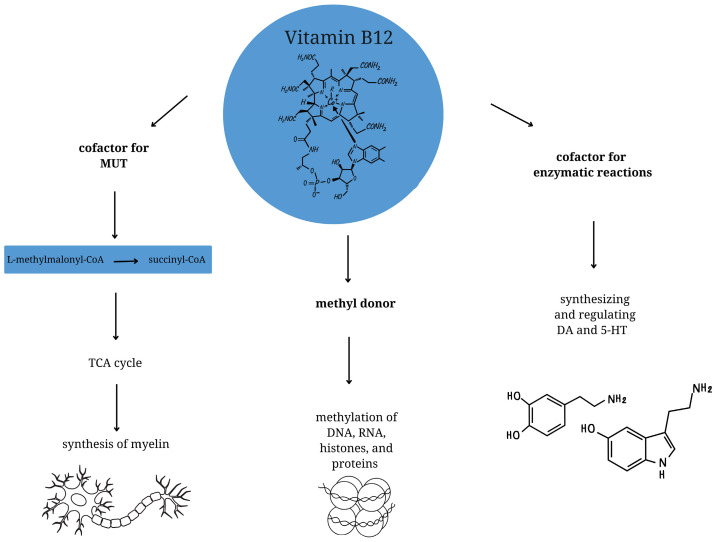
The role of vitamin B12 in neurodevelopment. Vitamin B12 acts as a cofactor for methylmalonyl-CoA mutase (MUT). MUT uses vitamin B12 in the form of adenosylcobalamin to facilitate the conversion of L-methylmalonyl-CoA to succinyl-CoA, which then enters the tricarboxylic acid (TCA) cycle, a key process in myelination. Cobalamin also plays a role as a methyl donor, which facilitates the correct methylation of DNA, RNA, histones and proteins. Vitamin B12 is involved in the synthesis and regulation of dopaminergic (DA) and serotonergic (5-HT) neurotransmitters.

Vitamin B12 is a complex compound comprising a central cobalt atom surrounded by a corrin ring and bound to various ligands [12]. At the cellular level, it functions as a cofactor for enzymatic reactions and is involved in synthesizing and regulating dopaminergic and serotonergic neurotransmitters [13]. Furthermore, it plays a crucial role in the production of hemoglobin, as it catalyzes the conversion of methylmalonic acid to succinyl-CoA, which is essential for optimal erythropoiesis [13,14]. With regard to neurodevelopment, vitamin B12 functions as a methyl donor in one-carbon metabolism, which facilitates the proper methylation of DNA, RNA, histones, and proteins [15]. Deficits in cobalamin at this level can result in a reduction in DNA synthesis and methylation levels [11]. Furthermore, vitamin B12 functions as a cofactor for methylmalonyl-CoA mutase (MUT) [16]. MUT employs cobalamin in the form of adenosylcobalamin to facilitate the conversion of L-methylmalonyl-CoA to succinyl-CoA, which subsequently enters the tricarboxylic acid (TCA) cycle. A lower level of vitamin B12 has been demonstrated to result in impaired MUT activity, which can in turn lead to defective synthesis of myelin, and thus to the development of neuropathy and nerve atrophy [17] (Figure 1).

It has been established that adequate levels of vitamin B12, with a particular emphasis on its active form, methylcobalamin, play a pivotal role in the homocysteine–methionine cycle. This role is attributed to the function of vitamin B12 as a cofactor for methionine synthase, which is an enzyme that catalyzes the conversion of homocysteine to methionine (Met) [18]. Met plays a pivotal role in the synthesis of S-adenosylmethionine (SAM), which serves as the primary source of methyl groups in methylation reactions. These reactions are of paramount importance for the regulation of gene expression, DNA stability, and the proper development of the central nervous system (CNS) and neurological functions [8]. Vitamin B12 deficits have been demonstrated to induce impairment in this metabolic pathway, consequently resulting in homocysteine accumulation, otherwise known as hyperhomocysteinemia. The condition has been identified as a risk factor for cardiovascular disease, neuropsychiatric disorders, and ASD, primarily due to the neurotoxic effects of homocysteine [19]. Research has demonstrated a correlation between insufficient levels of vitamin B12 and heightened homocysteine concentrations. This relationship has been implicated in the onset of neurodegenerative conditions, including dementia in the elderly population, and has been associated with an elevated risk of developing ASD [20]. Comparative analyses have demonstrated that children diagnosed with ASD exhibit considerably elevated levels of homocysteine and diminished levels of vitamin B12 and folic acid in comparison to children who do not meet the criteria for an ASD diagnosis [21]. These observations may be attributed to heightened oxidative stress and impaired DNA methylation [8].

## 4. The Relationship Between Vitamin B12 Deficits and Neurological Development

Numerous studies emphasize the necessity of maintaining sufficient levels of vitamin B12 from the beginning of pregnancy to facilitate its proper course and optimal neurological development of the infant. The Cruz-Rodriguez et al. study’s findings indicate that newborns whose mothers had an average serum cobalamin concentration of 312 to 408 pg/mL during pregnancy demonstrated superior motor skills, gross motor skills, language, and cognitive functions [22]. It has been demonstrated in other studies that infants born to mothers with vitamin B12 deficits during pregnancy exhibit substandard performance on cognitive assessments in comparison to infants born to mothers with adequate cobalamin levels. On the other hand, clear deficits in cognitive development were observed in the cohort of mothers who showed simultaneous lower levels of vitamin B12 and B6, which may indicate the additional contribution of deficits in other vitamins in the etiology of cognitive disorders in infants [23]. However, it should be noted that both of the above-mentioned studies were primarily observational, which excludes the possibility of drawing clear conclusions from them regarding the impact of lower levels of vitamin B12 in pregnant women on the development of neurodevelopmental dysfunction in children. However, some studies show no correlation between the total level of vitamin B12 or holotranscobalamin (the active form of vitamin B12) in the mother’s blood and the cognitive development of infants [24].

Similarly, five selected prospective cohort studies that were conducted in different countries and concerned the impact of maternal vitamin B12 supplementation on children’s cognitive development yielded inconclusive results. The studies analyzed data from mothers and their children, including indicators of cognitive, linguistic, and/or motor development. Some of them suggested a link between vitamin B12 deficits and poorer cognitive development in children; however, others did not confirm these observations [22,23,25,26,27]. Therefore, it is worth paying attention to the differentiating and converging factors in the selected group of publications. The first point of divergence is geographical diversity, which is important given that vitamin B12 deficit in both children and mothers can manifest itself with different frequencies depending on the region and the dietary patterns typical of the population that inhabits it. These studies were conducted in India, Singapore, the United Kingdom, and Spain. In addition, the researchers used different methods to assess cobalamin levels, and the specific time points at which measurements were taken varied considerably between studies. However, despite the numerous discrepancies, most studies, based on the sources provided, confirm the negative impact of low vitamin B12 levels in pregnant women on the subsequent neurological development of children.

Two randomized controlled trials evaluated the effect of vitamin B12 supplementation during pregnancy on children’s cognitive development, but the results also do not provide a clear answer. The first study did not show significant differences in the development of infants (9 months), although a correlation was noted between high homocysteine levels in the mother and poorer scores on some Bayley Scales of Infant Development III (BSID-III) subscales [28]. In the second study, children of mothers who supplemented with B12 performed better in expressive language at 30 months of age. According to a previous study, a significant correlation was identified between elevated homocysteine levels in the second and third trimesters of pregnancy and reduced development of expressive language and gross motor skills in children [29]. Vitamin B12 deficit is associated with hyperhomocysteinemia, which results in excessive accumulation of homocysteine in the body and consequent cell damage, and this condition is associated as a risk factor for neurodegenerative diseases [30]. Differences in the studies may be due to the discontinuation of supplementation after birth in the first study, the larger number of participants in the second, and the difficulty in assessing cognitive function in infants.

Despite these discrepancies, the studies suggest that long-term vitamin B12 supplementation during pregnancy may have a positive effect on the development of expressive language in children. Subsequent studies analyzing the effect of vitamin B12 deficits on the development of neurological disorders in children should also take homocysteine levels into account. Previous studies have shown that elevated homocysteine levels during pregnancy, like lower levels of vitamin B12, are risk factors for neural tube defects and related neurological complications [31]. Information regarding the level of vitamin B12 and the development of the nervous system is summarized in the Table 1.

## 5. Consequences of Excess Levels of Vitamin B12

In newborns whose mothers had adequate levels of vitamin B12, its stores at birth in serum are approximately 25 µg [32]. In the case of infants born to mothers with a cobalamin deficit, their endogenous reserves may be significantly reduced [33]. As described above, vitamin B12 is a vital nutrient for the optimal functioning of the nervous system and plays a pivotal role in DNA methylation processes [34,35]. Conversely, there is a paucity of evidence to suggest that an excess of cobalamin can also have adverse effects [36,37].

The study by Yuan et al. revealed a correlation between elevated (>95 percentile) maternal vitamin B12 levels and an increased risk of pregnancy complications, including intrahepatic cholestasis of pregnancy (ICP), preeclampsia (PE), and gestational diabetes mellitus (GDM). Additionally, high vitamin B12 levels have been shown to disrupt the natural development of the fetus, resulting in higher birth weight, and was associated with a higher risk of large-for-gestational-age (LGA) newborns [36]. This suggests that the optimal vitamin B12 and other vitamin levels for pregnant women need to be determined. This will allow for evidence-based recommendations on the use of this vitamin during pregnancy, so that the dose taken is safe for the fetus.

It is of significant importance to consider both deficits and excesses of vitamin B12 when administering vitamin B12 supplementation. It has been demonstrated that both infrequent (≤2 times per week) and frequent (>5 times per week) supplement intake is associated with an increased risk of ASD. Conversely, moderate multivitamin supplement intake (3–5 times per week) during pregnancy has been linked to a decreased risk of ASD. One of the key findings of this study was that maternal plasma vitamin B12 concentrations at birth exceeding 536.8 pmol/L were associated with a 2.5-fold increased risk of ASD in offspring. Notably, even higher maternal plasma vitamin B12 levels at the time of delivery (>600 pmol/L) were also found to correlate with an increased risk of ASD in children. While the 536.8 pmol/L threshold delineates the highest 10% of maternal B12 levels and is associated with a 2.5-fold increased risk, the >600 pmol/L threshold corresponds to an even narrower subgroup with an estimated 3.0-fold increase in ASD risk. Furthermore, the study highlighted an association between ASD risk and exceedingly high concentrations of both folate and vitamin B12 biomarkers (≥90th percentile) in newborns [38]. A notable limitation of the present study is the absence of longitudinal assessments of vitamin B12 levels in children beyond the neonatal period. While the study establishes an association between elevated maternal B12 concentrations at birth and an increased risk of ASD, it does not account for potential fluctuations in B12 status during later developmental stages. The findings of this research provide a foundation for future research aimed at identifying optimal levels of essential nutrients, such as cobalamin, that are crucial for normal fetal neurological development and child health after birth.

## 6. Impact of Maternal Vitamin B12 Levels During Pregnancy on ASD Risk in Offspring

The available literature contains only a limited number of studies that directly examine the relationship between vitamin B12 levels during pregnancy and the risk of developing autism spectrum disorder in offspring. The 3 months prior to conception and the first month of pregnancy have been identified as critical periods for the development of the fetal nervous system due to the closure of the neural tube. A disruption of this process can result in the development of autism, at least in some cases [39].

One study that evaluated the effect of vitamin B12 deficits on the development of autism is a long-term clinical-control study conducted by Sourander et al., which included 1558 children diagnosed with ASD and the same number of children in the control group. The results were inconclusive because no significant relationship was found between the level of vitamin B12 in the mother’s serum and the risk of autism spectrum disorder in the offspring. However, both elevated vitamin B12 levels (≥81st percentile) and reduced levels (<20th percentile) in the first trimester of pregnancy were associated with an increased risk of childhood autism in offspring [37]. The most important limitations of the study are the lack of routine vitamin B12 measurements in newborns and the fact that the study was conducted on people with strongly expressed ASD traits, which limits the possibility of generalizing the results.

On the other hand, Schmidt et al. have shown that prenatal intake of vitamins, including vitamin B12, in the preconception period can potentially reduce the risk of autism in a child, especially in mothers and children with specific variants of one-carbon metabolism genes (maternal *MTHFR* 677 TT, *CBS* rs234715 GT + TT, and child *COMT* 472 AA genotypes) [39]. However, the study has several limitations. The vitamin intake was based on the mothers’ self-assessment. Since their dietary habits were not analyzed, it cannot be ruled out that the information provided did not always correspond to the actual level of supplementation. Another limitation is the lack of vitamin B12 analysis in children.

In another study by Hollowood-Jones, a significant relationship was found only between low vitamin B12 levels and autism. Two to five years after giving birth, the metabolic profiles of mothers of children with autism spectrum disorder (ASD-M) were analyzed and compared with the profiles of mothers of typically developing children (TD-M). All participants had not consumed folate, vitamin B12, or multivitamin supplements for 2 months prior to sampling. The results show that ASD-M had some significantly different abnormalities associated with low vitamin B12 levels compared to TD-M. Moreover, vitamin B12 levels were significantly lower in the ASD-M group compared to the TD-M group, confirming the link between low vitamin B12 levels in mothers and autism [40].

Other studies of comparable significance suggest that, in addition to B12, other vitamins, including A, D, and K, may also contribute to an elevated risk of autism in offspring [41]. Chen et al. found a significant correlation between lower levels of vitamin D in the mother’s serum during the first trimester of pregnancy and an increased risk of autism spectrum disorder in the offspring [42]. However, the researchers did not confirm a link between cobalamin levels and the occurrence of autism. Noteworthy limitations of the study include its relatively small sample size, which hinders the reliability of the findings.

## 7. Effect of Vitamin B12 Deficits on Symptoms Severity in Children with Autism Spectrum Disorder

For a considerable period, scientists have been making intensive efforts to identify metabolic biomarkers that have the potential to serve as diagnostic tools for children with ASD, with the objective of differentiating them from healthy children [43]. The result of these efforts is a number of studies that point to low levels of vitamins, important for the development of the nervous system, as a potential differentiating factor. One study that examined this relationship is a comparative study by Zou et al. in which a reduced vitamin B12 level compared to normal was the differentiating factor between the group of children with ASD and the healthy group [44]. Li et al. obtained similar results, showing that both the vitamin B12 level was lower in children diagnosed with ASD and also that they scored higher on the Childhood Autism Rating Scale (CARS) compared to the control group [45]. Concurrently, a team of researchers led by Belardo et al. conducted a study to assess the potential impact of co-occurring deficits in vitamins B6, B9, and B12 on specific phenotypic characteristics associated with autism spectrum disorders. The researchers employed a metabolomic and methylomic approach to analyze urine samples from children with ASD who exhibited deficits in these vitamins [46]. Another particularly important study that analyzed the relationship between vitamin B12 levels and specific characteristics related to neurodevelopmental processes is the study conducted by Wu et al. The authors used the Gesell Developmental Scale to assess the functions that indicate the level of maturity of a child in the most important areas of his or her behavior. In children aged 2–4 years, serum vitamin B12 levels showed a positive correlation with language development quotients, while in the 4–6 age group, they correlated positively with adaptive development, motor skills, and social-personal behavior quotients [47]. While the aforementioned study suggests a potential correlation between vitamin B12 levels and autism, there are other studies, such as the observational study by Shi et al., which found no statistically significant difference in vitamin B12 levels between the ASD group and the typically developing control group. The study included a relatively small number of participants (132 children with ASD and 132 children with TD), which may have reduced the likelihood of detecting subtle differences in vitamin B12 levels between the groups [48]. Similarly, no significant differences in vitamin B12 levels were found between children with ASD and TD in another case-control study conducted in Haikou, China, by Guo et al. [49].

The studies discussed thus far have concentrated on examining the relationship between vitamin B12 levels and the severity of autism symptoms in groups of children with full-blown ASD. However, there is now a growing necessity to extend the scope of the study to encompass children exhibiting so-called “subdiagnostic” autism symptoms. The term refers to a situation in which a child exhibits some of the characteristics of ASD, but the presentation is not severe enough to meet the full diagnostic criteria for the disorder. This is the group that Arija et al. chose as their research target when assessing the intake of nutrients important for development among children with full autism and those with subdiagnostic autism symptoms. It turned out that the diet of both groups of children with neurodevelopmental disorders was poorer in terms of vitamin B12 content compared to children developing normally [50]. Information regarding the summary of scientific research on the impact of vitamin B12 deficiencies on the diagnosis of ASD and the severity of symptoms in children with ASD is presented in Table 2.

## 8. Possible Causes of Low Vitamin B12 Levels in ASD

Some studies show that children with autistic traits have relatively lower vitamin B12 levels compared to children without these traits, with the differences becoming more pronounced in later childhood. A nutrient-poor diet may be one of the factors responsible for cobalamin deficits. The dietary patterns of autistic individuals may affect their overall health and well-being, which is of particular importance in the context of their neurological development [51]. Vitamin B12 intake was shown to be significantly lower among children and adolescents with autistic traits compared to those without. The study’s authors suggest that the reduced intake of vitamin B12 may be related to the reduced consumption of animal products, fish, and legumes among those with autistic traits [52].

Another reason for a lack of cobalamin, which is often suggested by researchers, is malabsorption syndrome [53]. Erden et al. conducted a study that examined the association between malabsorption, vitamin B12 deficits, and the emergence of autism in children. The authors observed a substantial decrease in serum vitamin B12 levels in children diagnosed with autism. However, they did not establish a correlation between cobalamin levels and the presence of anti-phosphatidylserine antibodies (APCAs) [34]. Therefore, special attention should be paid to the diet of children with ASD, and monitoring of nutrient intake should be recommended throughout their development, rather than at specific points in time, as at each stage of development, the intake of adequate nutrients has a significant impact on the functioning of the nervous system.

## 9. Vitamin B12 Supplementation in ASD

An intriguing result emerged from a study conducted by Hendren et al., which sought to assess the potential of methyl-B12, a pivotal enhancer of methylation responses, in alleviating autism symptoms [54]. The study was based on the hypothesis that children with autism may have a limited ability to methylate DNA, as well as impaired antioxidant processes associated with the diagnosis of autism spectrum disorder [55]. A total of 57 autistic children aged 3 to 7 years with an IQ above 50 were randomly assigned to an 8-week treatment regimen of methyl-B12 at a dose of 75 µg/kg body weight or a placebo with saline solution. The clinical improvement achieved among children with ASD treated with methyl-B12, as compared to the control group, was positively correlated with an increase in methionine concentration in blood plasma, a decrease in S-adenosyl-l-homocysteine (SAH) concentration, and an improvement in the SAM to SAH ratio [54]. The results obtained indicate an increase in the ability to methylate DNA and cellular antioxidant potential under the influence of methyl-B12, as well as their potentially positive impact on the symptoms of autism.

On the other hand, a study conducted by Bertoglio et al. did not show an overall improvement in the group of children with autism receiving methylcobalamin, but 30% of the children showed a clinically significant change in behavior and an increase in glutathione (GSH) levels. These results suggest that methyl B12 supplementation may benefit children with autism, especially those with impaired methionine metabolism and/or increased oxidative stress. In Bertgolio’s study, all participants received both methylcobalamin and placebo for 6 weeks, but in a different order [56]. In contrast, the study by Hendren et al. was a parallel study, meaning that participants were randomly assigned to one of two groups: a group receiving methylcobalamin vitamin B12 or a placebo group [54]. Slight differences in the study design can have a significant impact on the results. In cross-over studies, there is a possibility of a carry-over effect, in which the effects of the initial treatment phase can influence the results of the subsequent phase. In parallel group studies, by implementing interventions in different groups simultaneously, this risk is reduced, increasing the reliability of the results. Both studies, despite different methodologies, indicate the potential effectiveness of methylcobalamin supplementation of vitamin B12 in some children with autism.

A meta-analysis evaluating the efficacy of cobalamin treatment in ASD demonstrated improvements in methylation capacity and the total glutathione redox ratio resulting from vitamin B12 supplementation. These improvements were associated with clinical improvements in ASD symptoms, including communication, interpersonal skills, and functioning in daily life. Additionally, improvements were observed in sleep, gastrointestinal symptoms, hyperactivity, tantrums, non-verbal IQ, eye contact, echolalia, stereotypies, anemia, and bedwetting. The most commonly observed adverse effects of the therapeutic intervention under examination were hyperactivity, irritability, sleep disturbances, aggression and behavioral deterioration. However, these were few and mild in intensity, and moreover, they did not differ significantly from those observed in the placebo group [57].

The majority of studies have centered on the role of methylcobalamin in alleviating symptoms of ASD, while data concerning the efficacy of other forms of vitamin B12 remains limited. The extant literature indicates that natural forms of cobalamin, such as methylcobalamin, adenosylcobalamin, and hydroxycobalamin, are preferred over synthetic cyanocobalamin due to their higher bioavailability and better safety profile [58]. As early as 1966, Chalmers et al. demonstrated that cyanocobalamin supplementation resulted in diminished tissue retention of vitamin B12 and augmented urinary excretion of its metabolites in comparison to other forms of cobalamin [59]. A potential rationale for the infrequent utilization of cyanocobalamin in research examining B12 supplementation in ASD may pertain to apprehension regarding the potential accumulation of cyanide in tissues following protracted administration of this particular vitamin form [60]. Consequently, although the metabolism of cyanocobalamin results in the release of only negligible amounts of cyanide, individuals with metabolic disorders, such as ASD, may be more vulnerable to its potential toxic effects. However, there is an absence of evidence supporting this hypothesis. Nevertheless, it is important to acknowledge that Obeida et al.’s analysis of various forms of vitamin B12, in terms of their efficacy in the prevention and treatment of deficiency, yielded insufficient evidence to unequivocally indicate an advantage for any of the forms in terms of bioavailability, biochemical effects, or clinical efficacy [61]. Nonetheless, clinical studies suggest the possibility of variations in the body’s response to different forms of cobalamin. For instance, a case report by Nashabat et al. documented the occurrence of acute anemia in two patients diagnosed with ASD and 2 TCN2 deficiency following cyanocobalamin supplementation. This anemia resolved subsequent to the conversion of this form to methylcobalamin [62].

## 10. Interaction of Vitamin B12 with Gut Microflora and Autism

Some studies indicate that autism may be a consequence of a distinct composition of gut microbiota (dysbiosis) compared to healthy individuals, through its impact on the gut–brain axis, especially since the gut microbiota produces nearly 40% of all human metabolites [63]. Meta-analysis showed that children with ASD exhibit dysbiosis compared to neurotypical children, with specific bacterial groups examined [64]. The microbiota of children with ASD, as evaluated in the study, exhibited an increased abundance of *Bacteroides*, *Parabacteroides*, *Clostridium*, *Faecalibacterium*, and *Phascolarctobacterium*, in comparison to a neurotypically developed control group. Conversely, a decreased abundance of *Coprococcus* and *Bifidobacterium* was observed. The alterations in the microbiota were linked not only to comorbid gastrointestinal issues but also to elevated autistic symptoms. However, this meta-analysis is not without limitations. The results of the studies are inconclusive and often contradictory. The groups analyzed were heterogeneous in terms of age, gender, methodology, and study design. Moreover, it was not possible to assess bacterial diversity at the species level. It is worth noting that there is no optimal and universal composition of the gut microbiota; rather, it is the balance and diversity in the bacterial population that is crucial for the proper functioning of the immune and nervous systems [64].

Furthermore, a growing body of research indicates that not only the infant’s microbiota, but also the mother’s gut dysbiosis during pregnancy may be important in the development of autism. The reason for this may be the state of maternal immune activation (MIA), which can potentially alter microglia function [65]. Dysbiosis may weaken the gut barrier, increasing the risk of inflammation and neurotoxic effects from bacterial metabolites [66]. Physiologically antimicrobial peptides produced by the intestinal epithelium are concentrated in the inner layer of the mucus covering the epithelium, where they destroy bacteria that have managed to penetrate the deeper layers of the mucus. This prevents damage to the bacterial cells of the microbiota that inhabit the outer layer of mucus. In addition, secretory IgA (sIgA) is more concentrated in the outer layer of mucus, protecting the intestinal microbiota from an immune response [67]. The intestinal barrier serves a protective function against the entry of pathogenic microorganisms and their metabolites, which could stimulate an immune response in the form of increased production of pro-inflammatory interleukins, including IL-1, IL-6, and IL-8. Studies have demonstrated that elevated levels of pro-inflammatory interleukins are a hallmark of children diagnosed with ASD, with increases in IL-1B, IL-6, IL-4, IFN-γ, and TGF-β observed [65].

Living in symbiosis with the human body, commensal microorganisms require a variety of vitamin combinations, with cobalamin serving as an essential enzyme cofactor for methionine synthesis, nucleotide metabolism, carbon and nitrogen metabolism, and other cellular processes. The synthesis and transport mechanisms utilized by different bacterial species vary [68]. As both humans and bacteria require exogenous cobalamins, it can be postulated that the gut microbiota may be in direct competition with its host for cobalamin [69]. It has also been shown that the production of vitamin B12 by bacteria colonizing the intestine enables them to modulate excitatory synaptic transmission [70]. Kang et al. used nematodes *C. elegans* with the unc-2/CaV2α(gf) mutation in presynaptic voltage-gated calcium channels as an animal model to study this relationship. Their goal was to recreate the conditions of increased excitatory transmission and imbalance between excitation and inhibition, which are likely to underlie autism. When the nematodes were given a medium containing bacteria that synthesize vitamin B12 (Comamonas aquatica, Pseudomonas putida), a significant reduction in hyperactivity was observed. This effect did not occur with bacterial strains that did not produce vitamin B12 [71].

On the other hand, it has been shown that cobalamin reduces cholinergic signaling in the nervous system by changing Met/SAM in the intestines, which consequently reduces the availability of free choline used by neurons to synthesize acetylcholine [71]. It is worth noting that previous studies have shown that low levels of acetylcholine may be a contributing factor to the onset of autism-related symptoms, and acetylcholinesterase inhibitors have emerged as a potential avenue for therapeutic intervention in ASD [72].

Two further animal studies that investigated the relationship between the microbiome, vitamin B12, and autism are particularly noteworthy. The first is an experimental study by Abujamel et al. on male Sprague Dawley rats. The authors investigated the effect of altering the intestinal microbiota—by administering *Bifidobacterium longum* (BF) and fecal microbiota transplantation (FT)—on vitamin B12 biosynthesis in the intestine. Supplementation with *Bifidobacterium longum* increased intestinal vitamin B12 biosynthesis and restored the behavioral phenotype of autistic rats to levels observed in the control group [73]. Equally important is the study by Alfawaz et al. on male Western Albino rats with propionic acid-induced autism (PPA) [74]. Inducing autistic traits in animals with propionic acid is a widely used research method for modeling the behavioral and neurological aspects of autism spectrum disorders in laboratory conditions. The study aimed to evaluate the effect of vitamin B12 supplementation on oxidative stress, lipid metabolism, and gut microbiota composition. Vitamin B12 supplementation showed beneficial effects in rats with PPA-induced autism. These effects included alleviation of oxidative stress, improvement of lipid metabolism, and modulation of the composition of intestinal microflora [74].

Conversely, a number of studies conducted among the human population have demonstrated that vitamin B12 supplementation in patients with vitamin B12 deficits results in a substantial increase in bacterial diversity, marked by the proliferation of *Firmicutes* and a decrease in *Bacteroidetes* [75]. In contrast, the results of a study conducted on exclusively breastfed infants with both normal and reduced vitamin B12 levels showed no statistically significant differences between the gut microbiota composition of the study group and the control group. The limitation of this study is the relatively short duration of vitamin B12 deficits in clinically asymptomatic infants, which may have prevented the observation of its impact on the gut microbiota. Furthermore, the cutoff point for deficiency, 203 pg/mL (150 pmol/L), was based on studies in older patients, which may not be directly applicable to infants [76].

Although knowledge about the influence of cobalamin on the microbiota and its composition on the level of this vitamin already provides us with important information regarding potential mechanisms involved in the etiopathogenesis of autistic disorders, many issues remain to be clarified. First of all, there is a lack of extensive research among children diagnosed with ASD. It seems equally important to study the bacterial flora in pregnant women, as maternal dysbiosis may play a significant role in the development of autism in children.

## 11. Conclusions

Vitamin B12 may play a key role in maintaining the health of the gut microbiota, whose imbalance is associated with the development of low-grade inflammation and changes in the gut–brain axis. Disruptions of this axis can affect the functioning of the nervous system and be potentially related to the development of autism spectrum disorder. In addition, cobalamin is involved in the metabolism of neurotransmitters such as serotonin and dopamine and in the myelination of neurons, which is crucial for proper brain development. People with ASD often show abnormalities in their intestinal microbiota and in their levels of B vitamins, including B12, which suggests that deficits in this vitamin may be one of the risk factors for ASD.

The results of studies conducted so far suggest that vitamin B12 may serve as a biomarker not only for predicting the course of the disease, but also for evaluating the effectiveness of therapy. Due to the inconsistency of the results obtained, additional studies, such as longitudinal studies, are required to determine whether there is a causal relationship between vitamin B12 levels and the severity of ASD symptoms, which will allow for monitoring changes over a longer period of time. It should be emphasized that long-term observation is essential to understand the dynamics of the relationship between vitamin B12 levels and ASD symptoms. Single-point measurements cannot fully account for the inherent variability in children’s health, nor do they account for potential external influences. Changes in vitamin levels and their impact on neurodevelopment may result from interactions with other factors, such as diet, environment, or therapeutic interventions. A longitudinal study would facilitate the tracking of these changes and increase the understanding of the potential impact of vitamin B12 levels on the neurological development of children with ASD at different stages of life.

The study of vitamin levels, including B12, should be part of a broader research initiative aimed at identifying biological factors that may affect the neurological development and behavior of children with ASD. An essential step in further research is the identification of specific biochemical markers to effectively select children who are most likely to benefit from vitamin B12 supplementation. This approach may facilitate earlier detection of disorders and contribute to the development of effective intervention strategies that can improve the quality of life of children affected by these disorders. Furthermore, the identification of vitamin B12 deficits in the ASD child population emphasizes the need for increased public awareness and education on prevention, which may help to reduce the prevalence of this disorder. In this context, it is necessary to encourage healthcare professionals to implement preventive measures, such as dietary modifications and the use of supplements, to provide effective support for children with ASD and their families.

## Figures and Tables

**Table 1:** Summary of studies on maternal vitamin B12 levels and infant neurological development.

Authors and Year	Study Type	Mother-Infant Population and Study Observation Period	Key Findings
Cruz-Rodríguez et al. (2023) [22]	Prospective Cohort Study (ECLIPSES Study)	Sample Size: 434 mother–infant pairs; 50.9% boys (221), 49.1% girls (213). Observation Points: 12th week of pregnancy, 36th week of pregnancy, 40 days postpartum (neurodevelopmental assessment).	Adequate maternal B12 levels (312–408 pg/mL) in the first trimester were linked to higher motor, gross motor, language, and cognitive scores.
Lai et al. (2019) [23]	Prospective Cohort Study (GUSTO Study)	Sample Size: 443 mother–infant pairs from the GUSTO cohort; 235 boys and 208 girls. Observation Points: 26–28 weeks of pregnancy (maternal plasma vitamin B12 levels), infant cognitive development assessment at 24 months.	Maternal B12 deficiency during pregnancy was associated with lower cognitive function scores in 24-month-old infants.
Wu et al. (2012) [24]	Prospective Study	Sample Size: 154 mother-infant pairs. Observation Points: 16th week of pregnancy, 36th week of pregnancy, infant neurological assessment at 18 months.	No significant association was found between maternal folate, total B12 levels and infant development.
Bhate et al. (2012) [25]	Prospective Longitudinal Cohort Study	Sample Size: 123 mother–infant pairs (62 girls, 61 boys). Observation Points: 28th week of pregnancy, 34th week of pregnancy, at birth, 2 years postpartum.	Maternal B12 deficiency during pregnancy is linked to poorer cognitive and social development in 2-year-olds.
Golding et al. (2021) [26]	Cohort Study (ALSPAC)	Sample Size: 14,541 pregnancies initially; 14,062 live births; 13,988 children survived the first year; 12,095 women included in vitamin B12 dietary analysis. Observation Points: Cognitive assessments at 8 and 15 years, speech and language at 24 months, 38 months, 6 years, and 15 years, mathematical ability at 4, 6, and 8 school years, reading and writing at 7, 9, and 13–14 years.	Low maternal B12 intake during pregnancy was associated with adverse child development outcomes, particularly in certain speech and math skills.
Bonilla et al. (2012) [27]	Mendelian Randomization Study (ALSPAC Cohort Data Analysis)	Sample Size: Over 14,000 pregnant women enrolled; 10,851 assessed for dietary vitamin B12 intake; 6259 children assessed for cognitive function; 310 umbilical cord blood samples analyzed for vitamin B12 levels. Observation Points: 32nd week of pregnancy (maternal dietary vitamin B12 intake), birth (umbilical cord blood vitamin B12 levels in a subset), cognitive function assessment at 8 years and 7 months.	Positive association was found between maternal dietary B12 intake and child’s IQ.
Srinivasan et al. (2017) [28]	RCT	Sample Size: 366 pregnant women enrolled; 178 infants assessed for development at 9 months. Observation Points: Before 14th week of pregnancy (maternal enrollment), throughout pregnancy (vitamin B12 supplementation), 6 weeks postpartum (continued supplementation), infant developmental assessment at 9 months.	Maternal B12 supplementation during pregnancy had no significant impact on infants’ cognitive development at 9 months.
Thomas et al. (2019) [29]	Placebo-Controlled RCT (Follow-up of Srinivasan et al. 2017 [28])	Sample Size: 218 children; 57 boys (50%) in the intervention group, 50 boys (48.07%) in the placebo group. Pregnant women who supplemented with vitamin B12 during pregnancy and early lactation. Observation Points: Cognitive assessment at 30 months, previous assessment at 9 months (Srinivasan et al. 2017 [28]).	Maternal B12 supplementation during pregnancy was associated with higher expressive language scores in 30-month-old children.

Abbreviations used in the table: Avon Longitudinal Study of Parents and Children (ALSPAC), Randomized Controlled Trial (RCT), Growing Up in Singapore Towards Healthy Out-Comes (GUSTO).

**Table 2:** Summary of scientific studies on the impact of vitamin B12 deficiencies on ASD diagnosis and symptom severity in children with ASD.

Authors and Year	Study Population	Methods for Assessing ASD Symptoms	Vitamin B12 Levels in Children with ASD	Symptom Severity/Key Findings
Zou et al. (2019) [44]	89 ASD cases and 89 control subjects; no data on ASD diagnostic criteria.	The severity of ASD symptoms was not assessed.	Vitamin B12 was one of 10 metabolites that significantly differed between ASD and control groups (lower B12 levels in ASD).	The severity of ASD symptoms was not assessed.
Li et al. (2024) [45]	50 ASD cases and 50 control subjects, aged 3–12 years. ASD diagnosis based on ADI-R and ADOS.	CARS	Children with ASD had significantly lower serum vitamin B12 levels (502.69 pmol/L) compared to the control group (626.41 pmol/L).	A significant negative correlation was found between vitamin B12 levels and CARS scores, suggesting that lower vitamin B12 levels may be associated with greater ASD symptom severity.
Belardo et al. (2019) [46]	60 ASD cases and 60 control subjects, aged 3–8 years. The male-to-female ratio was 42:18 in both groups. ASD diagnosis was based on DSM-5 criteria.	The severity of ASD symptoms was not assessed.	Lowered (based on elevated urinary methylmalonic acid levels).	The severity of ASD symptoms was not assessed—only an indirect association suggested through methylation disturbances and increased homocysteine levels, which may affect the ASD phenotype.
Wu et al. (2024) [47]	324 ASD cases, 318 control subjects, aged 2–6 years; 257 boys and 67 girls in the ASD group, 157 boys and 161 girls in the control group; ASD diagnosis based on DSM-5 criteria.	SRS, CARS, GDS	Children with ASD had significantly lower serum vitamin B12 levels (667.9 pmol/L) compared to healthy children (812.2 pmol/L).	Serum vitamin B12 levels were not significantly associated with autism symptom severity. In the 2- to < 4-year-old ASD group, B12 levels showed a positive correlation with language development. In the 4- to 6-year-old ASD group, B12 levels were positively correlated with adaptive, motor, and social-personal development.
Shi et al. (2024) [48]	132 ASD cases and 132 control subjects; 119 boys and 13 girls in both groups. ASD diagnosis based on DSM-5 criteria and ADOS.	The severity of ASD symptoms was not assessed.	No significant differences in average vitamin B12 levels were found between children with ASD and typically developing children in the studied population.	The direct impact of vitamin B12 deficiency on ASD symptom severity was not analyzed.
Guo et al. (2020) [49]	274 ASD cases, children aged 2–7 years (mean age 4.06 ± 1.13), with 236 boys and 38 girls. 97 control subjects (mean age 4.24 ± 1.20), with 50 boys and 47 girls. No data on ASD diagnostic criteria.	ABC, SRS, GDS	Vitamin B12 levels were found in children with ASD, but no significant correlations with symptom severity were observed in the studied population.	No statistically significant direct correlations were found between vitamin B12 levels and ASD symptoms.
Arija et al. (2022) [50]	77 ASD cases, 40 with subdiagnostic autistic traits, and 333 control subjects. No data on sex ratio or ASD diagnostic criteria.	The severity of ASD symptoms was not assessed.	Children with ASD and subclinical autistic traits had slightly lower vitamin B12 intake compared to typically developing children—no data on blood vitamin B12 levels.	Few differences in nutrient intake were found between the ASD and TD groups.

Abbreviations used in the table: Autism Spectrum Disorder (ASD), Autism Behavior Checklist (ABC), Social Responsiveness Scale (SRS), Gesell Developmental Scale (GDS), Childhood Autism Rating Scale (CARS), Autism Diagnostic Observation Schedule (ADOS), Diagnostic and Statistical Manual of Mental Disorders, 5th Edition (DSM-5), Autism Diagnostic Interview-Revised (ADI-R).

## References

[1] Hanna M., Jaqua E., Nguyen V., Clay J. (2022). B Vitamins: Functions and Uses in Medicine. Perm. J..

[2] Krishnaveni G.V., Hill J.C., Veena S.R., Bhat D.S., Wills A.K., Karat C.L.S., Yajnik C.S., Fall C.H.D. (2009). Low plasma vitamin B12 in pregnancy is associated with gestational ‘diabesity’ and later diabetes. Diabetologia.

[3] Knight B.A., Shields B.M., Brook A., Hill A., Bhat D.S., Hattersley A.T., Yajnik C.S. (2015). Lower Circulating B12 Is Associated with Higher Obesity and Insulin Resistance during Pregnancy in a Non-Diabetic White British Population. PLoS ONE.

[4] Health Canada (1999). Nutrition for a Healthy Pregnancy: National Guidelines for the Childbearing Years.

[5] Hibbeln J.R., SanGiovanni J.P., Golding J., Emmett P.M., Northstone K., Davis J.M., Schuckit M., Heron J. (2017). Meat Consumption During Pregnancy and Substance Misuse Among Adolescent Offspring: Stratification of *TCN2* Genetic Variants. Alcohol. Clin. Exp. Res..

[6] Hunt A., Harrington D., Robinson S. (2014). Vitamin B12 deficiency. BMJ.

[7] Kennedy D.O. (2016). B Vitamins and the Brain: Mechanisms, Dose and Efficacy—A Review. Nutrients.

[8] Yektaş Ç., Alpay M., Tufan A.E. (2019). Comparison of serum B12, folate and homocysteine concentrations in children with autism spectrum disorder or attention deficit hyperactivity disorder and healthy controls. Neuropsychiatr. Dis. Treat..

[9] Tan Y., Zhou L., Gu K., Xie C., Wang Y., Cha L., Wu Y., Wang J., Song X., Chen X. (2023). Correlation between Vitamin B12 and Mental Health in Children and Adolescents: A Systematic Review and Meta-analysis. Clin. Psychopharmacol. Neurosci..

[10] Finkelstein J.L., Layden A.J., Stover P.J. (2015). Vitamin B-12 and Perinatal Health. Adv. Nutr. Int. Rev. J..

[11] Zeeshan F., Bari A., Farhan S., Jabeen U., Rathore A.W. (2017). Correlation between maternal and childhood VitB12, folic acid and ferritin levels. Pak. J. Med. Sci..

[12] Pepper M.R., Black M.M. (2011). B12 in fetal development. Semin. Cell Dev. Biol..

[13] Young L.M., Pipingas A., White D.J., Gauci S., Scholey A. (2019). A Systematic Review and Meta-Analysis of B Vitamin Supplementation on Depressive Symptoms, Anxiety, and Stress: Effects on Healthy and ‘At-Risk’ Individuals. Nutrients.

[14] Bellazzi F., Bertolaso M. (2024). Emergence in Complex Physiological Processes: The Case of Vitamin B12 Functions in Erythropoiesis. Systems.

[15] Zeisel S. (2017). Choline, Other Methyl-Donors and Epigenetics. Nutrients.

[16] Halczuk K., Kaźmierczak-Barańska J., Karwowski B.T., Karmańska A., Cieślak M. (2023). Vitamin B12—Multifaceted In Vivo Functions and In Vitro Applications. Nutrients.

[17] Wongkittichote P., Cunningham G., Summar M.L., Pumbo E., Forny P., Baumgartner M.R., Chapman K.A. (2019). Tricarboxylic acid cycle enzyme activities in a mouse model of methylmalonic aciduria. Mol. Genet. Metab..

[18] Uche E.I., Akinbami A.A., Bamiro R., Adeyemi I.O., Ibrahim N. (2022). Evaluation of serum folate, vitamin B12 and homocysteine among blood donors in Lagos State University teaching hospital, Lagos, Nigeria. Int. J. Sci. Rep..

[19] Al-Beltagy S.B., El-Serogy H.A., Elaziz S.A.E.A., Al-Gohary T.M. (2022). A Study of Plasma Homocysteine Level in Children with Autism. Asian J. Pediatr. Res..

[20] Walkiewicz K.W., Dzięgielewska-Gęsiak S., Fatyga E., Gętek M., Kozieł P., Muc-Wierzgoń M., Kokot T., Nowakowska-Zajdel E. (2015). Cognitive impairment in elderly patients considering concentration of hemoglobin and vitamin B 12—Preliminary report. Fam. Med. Prim. Care Rev..

[21] Nesa A., Sultana G.S. (2023). Study of Homocysteine, Vitamin B12 and Folate in children with autism spectrum disorder: Vitamin B12 and Folate in children with autism spectrum disorder. Bangladesh Med. Res. Counc. Bull..

[22] Cruz-Rodríguez J., Díaz-López A., Canals-Sans J., Arija V. (2023). Maternal Vitamin B12 Status during Pregnancy and Early Infant Neurodevelopment: The ECLIPSES Study. Nutrients.

[23] Lai J.S., Ayob M.N.M., Cai S., Quah P.L., Gluckman P.D., Shek L.P., Yap F., Tan K.H., Chong Y.S., Godfrey K.M. (2019). Maternal plasma vitamin B12 concentrations during pregnancy and infant cognitive outcomes at 2 years of age. Br. J. Nutr..

[24] Wu B.T.F., Dyer R.A., King D.J., Richardson K.J., Innis S.M. (2012). Early Second Trimester Maternal Plasma Choline and Betaine Are Related to Measures of Early Cognitive Development in Term Infants. PLoS ONE.

[25] Bhate V.K., Joshi S.M., Ladkat R.S., Deshmukh U.S., Lubree H.G., Katre P.A., Bhat D.S., Rush E.C., Yajnik C.S. (2011). Vitamin B12 and folate during pregnancy and offspring motor, mental and social development at 2 years of age. J. Dev. Orig. Health Dis..

[26] Golding J., Gregory S., Clark R., Iles-Caven Y., Ellis G., Taylor C.M., Hibbeln J. (2021). Maternal prenatal vitamin B12 intake is associated with speech development and mathematical abilities in childhood. Nutr. Res..

[27] Bonilla C., Lawlor D.A., Taylor A.E., Gunnell D.J., Ben–Shlomo Y., Ness A.R., Timpson N.J., Pourcain B.S., Ring S.M., Emmett P.M. (2012). Vitamin B-12 Status during Pregnancy and Child’s IQ at Age 8: A Mendelian Randomization Study in the Avon Longitudinal Study of Parents and Children. PLoS ONE.

[28] Srinivasan K., Thomas T., Kapanee A.R.M., Ramthal A., Bellinger D.C., Bosch R.J., Kurpad A.V., Duggan C. (2016). Effects of maternal vitamin B12 supplementation on early infant neurocognitive outcomes: A randomized controlled clinical trial. Matern. Child Nutr..

[29] Thomas S., Thomas T., Bosch R.J., Ramthal A., Bellinger D.C., Kurpad A.V., Duggan C.P., Srinivasan K. (2018). Effect of Maternal Vitamin B12 Supplementation on Cognitive Outcomes in South Indian Children: A Randomized Controlled Clinical Trial. Matern. Child Health J..

[30] Waligóra A., Waligóra S., Kozarska M., Damasiewicz-Bodzek A., Gorczyca P., Tyrpień-Golder K. (2019). Autism spectrum disorder (ASD)—Biomarkers of oxidative stress and methylation and transsulfuration cycle. Psychiatr. Polska.

[31] Gąsiorowska D., Korzeniowska K., Jabłecka A. (2009). Ocena Stężenia Homocysteiny u Chorych z Miażdżycowym Niedokrwieniem Kończyn Dolnych (Evaluation of the Homocysteine Concentration in the Patients with Atheromatous Ischemia of Lower Extremities). https://www.akademiamedycyny.pl/wp-content/uploads/2016/05/200901_Farmacja_004.pdf.

[32] McPhee A.J., Davidson G.P., Leahy M., Beare T. (1988). Vitamin B12 deficiency in a breast fed infant. Arch. Dis. Child..

[33] Dror D.K., Allen L.H. (2008). Effect of vitamin B12 deficiency on neurodevelopment in infants: Current knowledge and possible mechanisms. Nutr. Rev..

[34] Erden S., İleri B.A., Çelikkol Ç.S., Nalbant K., Kılınç I., Yazar A. (2021). Serum B12, homocysteine, and anti-parietal cell antibody levels in children with autism. Int. J. Psychiatry Clin. Pract..

[35] Li C.-X., Liu Y.-G., Che Y.-P., Ou J.-L., Ruan W.-C., Yu Y.-L., Li H.-F. (2021). Association Between MTHFR C677T Polymorphism and Susceptibility to Autism Spectrum Disorders: A Meta-Analysis in Chinese Han Population. Front. Pediatr..

[36] Yuan X., Han X., Zhou W., Long W., Wang H., Yu B., Zhang B. (2022). Association of folate and vitamin B12 imbalance with adverse pregnancy outcomes among 11,549 pregnant women: An observational cohort study. Front. Nutr..

[37] Sourander A., Silwal S., Surcel H.-M., Hinkka-Yli-Salomäki S., Upadhyaya S., McKeague I.W., Cheslack-Postava K., Brown A.S. (2023). Maternal Serum Vitamin B12 during Pregnancy and Offspring Autism Spectrum Disorder. Nutrients.

[38] Raghavan R., Riley A.W., Volk H., Caruso D., Hironaka L., Sices L., Hong X., Wang G., Ji Y., Brucato M. (2017). Maternal Multivitamin Intake, Plasma Folate and Vitamin B_12_ Levels and Autism Spectrum Disorder Risk in Offspring. Paediatr. Perinat. Epidemiol..

[39] Schmidt R.J., Hansen R.L., Hartiala J., Allayee H., Schmidt L.C., Tancredi D.J., Tassone F., Hertz-Picciotto I. (2011). Prenatal Vitamins, One-carbon Metabolism Gene Variants, and Risk for Autism. Epidemiology.

[40] Hollowood-Jones K., Adams J.B., Coleman D.M., Ramamoorthy S., Melnyk S., James S.J., Woodruff B.K., Pollard E.L., Snozek C.L., Kruger U. (2020). Altered metabolism of mothers of young children with Autism Spectrum Disorder: A case control study. BMC Pediatr..

[41] Ribeiro R., Nicoli J.R., Santos G., Lima-Santos J. (2019). Impact of vitamin deficiency on microbiota composition and immunomodulation: Relevance to autistic spectrum disorders. Nutr. Neurosci..

[42] Chen J., Xin K., Wei J., Zhang K., Xiao H. (2016). Lower maternal serum 25(OH) D in first trimester associated with higher autism risk in Chinese offspring. J. Psychosom. Res..

[43] Khemakhem A.M., Frye R.E., El-Ansary A., Al-Ayadhi L., Ben Bacha A. (2017). Novel biomarkers of metabolic dysfunction is autism spectrum disorder: Potential for biological diagnostic markers. Metab. Brain Dis..

[44] Zou M., Sun C., Liang S., Sun Y., Li D., Li L., Fan L., Wu L., Xia W. (2019). Fisher discriminant analysis for classification of autism spectrum disorders based on folate-related metabolism markers. J. Nutr. Biochem..

[45] Li H., Dang Y., Yan Y. (2024). Serum interleukin-17 A and homocysteine levels in children with autism. BMC Neurosci..

[46] Belardo A., Gevi F., Zolla L. (2019). The concomitant lower concentrations of vitamins B6, B9 and B12 may cause methylation deficiency in autistic children. J. Nutr. Biochem..

[47] Wu Y., Yang T., Chen H.-Y., Long D., Xiang X.-L., Feng Y.-R., Wei Q.-H., Chen J., Li T.-Y. (2024). Serum folate and vitamin B12 levels and their association with neurodevelopmental features in preschool children with autism spectrum disorder. Chin. J. Contemp. Pediatr..

[48] Shi A., Liu D., Wu H., Zhu R., Deng Y., Yao L., Xiao Y., Lorimer G.H., Ghiladi R.A., Xu X. (2024). Serum binding folate receptor autoantibodies lower in autistic boys and positively-correlated with folate. Biomed. Pharmacother..

[49] Guo M., Li L., Zhang Q., Chen L., Dai Y., Liu L., Feng J., Cai X., Cheng Q., Chen J. (2018). Vitamin and mineral status of children with autism spectrum disorder in Hainan Province of China: Associations with symptoms. Nutr. Neurosci..

[50] Arija V., Esteban-Figuerola P., Morales-Hidalgo P., Jardí C., Canals-Sans J. (2022). Nutrient intake and adequacy in children with autism spectrum disorder: EPINED epidemiological study. Autism.

[51] Al-Beltagi M. (2024). Nutritional management and autism spectrum disorder: A systematic review. World J. Clin. Pediatr..

[52] Tsujiguchi H., Miyagi S., Nguyen T.T.T., Hara A., Ono Y., Kambayashi Y., Shimizu Y., Nakamura H., Suzuki K., Suzuki F. (2020). Relationship between Autistic Traits and Nutrient Intake among Japanese Children and Adolescents. Nutrients.

[53] Guéant J.L., Guéant-Rodriguez R.M., Alpers D.H. (2022). Vitamin B12 absorption and malabsorption. Vitam. Horm..

[54] Hendren R.L., James S.J., Widjaja F., Lawton B., Rosenblatt A., Bent S. (2016). Randomized, Placebo-Controlled Trial of Methyl B12 for Children with Autism. J. Child Adolesc. Psychopharmacol..

[55] Melnyk S., Fuchs G.J., Schulz E., Lopez M., Kahler S.G., Fussell J.J., Bellando J., Pavliv O., Rose S., Seidel L. (2011). Metabolic Imbalance Associated with Methylation Dysregulation and Oxidative Damage in Children with Autism. J. Autism Dev. Disord..

[56] Bertoglio K., Jill James S., Deprey L., Brule N., Hendren R.L. (2010). Pilot Study of the Effect of Methyl B12 Treatment on Behavioral and Biomarker Measures in Children with Autism. J. Altern. Complement. Med..

[57] Rossignol D.A., Frye R.E. (2021). The Effectiveness of Cobalamin (B12) Treatment for Autism Spectrum Disorder: A Systematic Review and Meta-Analysis. J. Pers. Med..

[58] Paul C., Brady D.M. (2017). Comparative Bioavailability and Utilization of Particular Forms of B12 Supplements with Potential to Mitigate B12-related Genetic Polymorphisms. Integr. Med. A Clin. J..

[59] Chalmers J.M., Shinton N. (1965). Comparison of hydroxocobalamin and cyanocobalamin in the treatment of pernicious anaemia. Lancet.

[60] Gatica-Domínguez G., Rothenberg S.J., Torres-Sánchez L., Schnaas M.d.L., Schmidt R.J., López-Carrillo L. (2018). Child dietary intake of folate and vitamin B12 and their neurodevelopment at 24 and 30 months of age. Salud Publica Mex..

[61] Obeid R., Fedosov S.N., Nexo E. (2015). Cobalamin coenzyme forms are not likely to be superior to cyano- and hydroxyl-cobalamin in prevention or treatment of cobalamin deficiency. Mol. Nutr. Food Res..

[62] Nashabat M., Maegawa G., Nissen P.H., Nexo E., Al-Shamrani H., Al-Owain M., Alfadhel M. (2017). Long-term Outcome of 4 Patients with Transcobalamin Deficiency Caused by 2 Novel TCN2 Mutations. J. Pediatr. Hematol..

[63] Vuong H.E., Hsiao E.Y. (2017). Emerging Roles for the Gut Microbiome in Autism Spectrum Disorder. Biol. Psychiatry.

[64] Iglesias-Vázquez L., Riba G.V.G., Arija V., Canals J. (2020). Composition of Gut Microbiota in Children with Autism Spectrum Disorder: A Systematic Review and Meta-Analysis. Nutrients.

[65] Suprunowicz M., Tomaszek N., Urbaniak A., Zackiewicz K., Modzelewski S., Waszkiewicz N. (2024). Between Dysbiosis, Maternal Immune Activation and Autism: Is There a Common Pathway?. Nutrients.

[66] Kinashi Y., Hase K. (2021). Partners in Leaky Gut Syndrome: Intestinal Dysbiosis and Autoimmunity. Front. Immunol..

[67] Fakhoury H.M.A., Kvietys P.R., AlKattan W., Al Anouti F.A., Elahi M.A., Karras S.N., Grant W.B. (2020). Vitamin D and intestinal homeostasis: Barrier, microbiota, and immune modulation. J. Steroid Biochem. Mol. Biol..

[68] Putnam E.E., Goodman A.L. (2020). B vitamin acquisition by gut commensal bacteria. PLoS Pathog..

[69] Degnan P.H., Taga M.E., Goodman A.L. (2014). Vitamin B 12 as a Modulator of Gut Microbial Ecology. Cell Metab..

[70] Gao R., Penzes P. (2015). Common Mechanisms of Excitatory and Inhibitory Imbalance in Schizophrenia and Autism Spectrum Disorders. Curr. Mol. Med..

[71] Kang W.K., Araya A., Fox B.W., Thackeray A., Schroeder F.C., Walhout A.J., Alkema M.J. (2022). Vitamin B12 produced by gut bacteria modulates excitatory neurotransmission. bioRxiv.

[72] Ure A., Cox G.R., Haslam R., Williams K. (2023). Acetylcholinesterase inhibitors for autistic spectrum disorders. Cochrane Database Syst. Rev..

[73] Abujamel T.S., Al-Otaibi N.M., Abuaish S., AlHarbi R.H., Assas M.B., Alzahrani S.A., Alotaibi S.M., El-Ansary A., Aabed K. (2022). Different Alterations in Gut Microbiota between *Bifidobacterium longum* and Fecal Microbiota Transplantation Treatments in Propionic Acid Rat Model of Autism. Nutrients.

[74] Alfawaz H., Bhat R.S., Al-Mutairi M., Alnakhli O.M., Al-Dbass A., AlOnazi M., Al-Mrshoud M., Hasan I.H., El-Ansary A. (2018). Comparative study on the independent and combined effects of omega-3 and vitamin B12 on phospholipids and phospholipase A2 as phospholipid hydrolyzing enzymes in PPA-treated rats as a model for autistic traits. Lipids Health Dis..

[75] Guetterman H.M., Huey S.L., Knight R., Fox A.M., Mehta S., Finkelstein J.L. (2021). Vitamin B-12 and the Gastrointestinal Microbiome: A Systematic Review. Adv. Nutr. Int. Rev. J..

[76] Boran P., Baris H.E., Kepenekli E., Erzik C., Soysal A., Dinh D.M. (2019). The impact of vitamin B12 deficiency on infant gut microbiota. Eur. J. Pediatr..

